# Efficacy, Immunogenicity and Safety of COVID-19 Vaccines: A Systematic Review and Meta-Analysis

**DOI:** 10.3389/fimmu.2021.714170

**Published:** 2021-10-11

**Authors:** Nadim Sharif, Khalid J. Alzahrani, Shamsun Nahar Ahmed, Shuvra Kanti Dey

**Affiliations:** ^1^ Department of Microbiology, Jahangirnagar University, Dhaka, Bangladesh; ^2^ Department of Clinical Laboratories Sciences, College of Applied Medical Sciences, Taif University, Taif, Saudi Arabia

**Keywords:** COVID-19 vaccines, efficacy, safety, immunogenicity, meta-analysis

## Abstract

There is a significant research gap in meta-analysis on the efficacy and safety of coronavirus disease 2019 (COVID-19) vaccines. This study analyzed the efficacy of COVID-19 vaccines. Published phase I, phase II, and phase III trials analyzing safety and immunogenicity and phase III randomized clinical trials evaluating the efficacy of COVID-19 vaccines were included. We searched MEDLINE, Scopus, and The Lancet for published articles evaluating the relative reduction in COVID-19 risk after vaccination. Selected literatures were published between December 15, 2019 and May 15, 2021 on the safety, efficacy, and immunogenicity of COVID-19 vaccines. This meta-analysis included studies that confirmed cases of COVID-19 using reverse transcriptase polymerase chain reaction. This study detected 8,926 eligible research articles published on COVID-19 vaccines. Of these, 25 studies fulfilled the inclusion criteria. Among the selected articles, 19 randomized clinical trials, 2 non-randomized clinical trials, and 3 observational studies were analyzed. Seven (28%) studies were included in the meta-analysis. The efficacy of the adenovirus vector vaccine was 73% (95% CI = 69–77) and that of the messenger RNA (mRNA) vaccine was 85% (95% CI = 82–88) in participants aged ≥18 years. There are no reports of clinical trials in participants aged under 16 years. The production of neutralizing antibodies against receptor-binding domains (RBDs) of severe acute respiratory syndrome coronavirus 2 (SARS-CoV-2) in >90% of the vaccinated samples was reported within 0–30 days of the first or the second dose of the vaccine. Pain at the injection site was the most common local symptom in people receiving mRNA vaccines (29%–85% of participants). Fever (0.2%–95%) was the most prevalent in people receiving adenovirus vector vaccines, and fatigue (8.4%–55%) was the most common side effect in people receiving the mRNA vaccines. Studies suggest that mRNA vaccines and adenovirus vector vaccines can provide moderate to high protection against COVID-19 infection in people over 18 years. Evidence of the long-term protection of the vaccines in people aged under 16 years against the multiple variants of COVID-19 are limited. This study will provide an integrated evaluation on the efficacy, safety, and immunogenicity of the COVID-19 vaccines.

## Introduction

A novel species of coronavirus, namely, severe acute respiratory syndrome coronavirus 2 (SARS-CoV-2), has set out the coronavirus disease 2019 (COVID-19) pandemic from December 2020 (1). About 150 million cases and 3.2 million fatalities associated with COVID-19 have been confirmed globally (2, 3). The odds of acquiring health issues are higher in the elderly, healthcare workers, persons with comorbidities, and those who live in areas with high community transmission (4–8). According to recent studies, children and younger adults are also becoming infected with COVID-19 and are having serious health problems (4). The majority of the world’s population is still uninfected. However, the number of illnesses and fatalities are continuously increasing (2, 3). If adequate preventive actions are not performed quickly, COVID-19 will have serious and long-term medical, social, economic, and mental effects (9, 10).

Effective vaccines are one of the most significant preventive measures to contain infectious diseases (10). Immunization against COVID-19 *via* vaccines will not only prevent the spread of the virus but will also limit the serious health consequences of the pandemic (9, 10). Several vaccine candidates have been tested and found to be effective and safe against COVID-19. Since December 2020, different countries have begun mass vaccinations and targeted population vaccinations. Two messenger RNA (mRNA) vaccines, three adenovirus vector vaccines, four inactivated vaccines, and two protein subunit vaccines have been approved for use against COVID-19 at the national and international levels (9, 11, 12). The safety and efficacy of these vaccine candidates were evaluated in laboratory studies, randomized clinical trials, and observational studies before they were approved for emergency or full use (9, 10).

Vaccines against COVID-19 were developed and utilized in a relatively short period of time compared to other vaccines. As a result, the efficacy, safety, and side effects of the vaccines against COVID-19 require continuous and extensive surveillance and research (9, 10). Three factors should be considered to assess the effectiveness and safety of the COVID-19 vaccines for long-term prevention: the emergence of new SARS-CoV-2 variants with altered infection capacity and immune neutralization properties, the side effects of the vaccine in different socio-demographic settings, and the longevity of the produced antibodies against the virus (9, 10, 13–15). New variants, namely, alpha (B.1.1.7), beta (B.1.351), gamma (P.1), iota (B.1.526), epsilon (B.1.429), and delta (B.1.617.2), have emerged and have been transmitted to different countries within short periods of time (14, 15). As a result, randomized controlled trials and observational studies are needed to confirm the efficacy of the existing vaccines against the newly emerged variants (13, 15).

One of the most important predictors of vaccine acceptance in recipients is the safety of the available vaccines (16–20). In the majority of randomized clinical and observational studies, local and systematic reactions in recipients after vaccination have been reported in the mRNA vaccines, adenovirus vector vaccines, inactivated virus vaccines, and the protein subunit vaccines (16, 17, 21–26). Both single-dose and double-dose vaccines had side effects.

The most common local reactions were pain, erythema, swelling, and lymphadenopathy at the injection site, while headache, fatigue, myalgia, and nausea were the most common systemic side effects of the COVID-19 vaccines (27–35). Serious grade 3 consequences possibly associated with COVID-19 were detected at a very low frequency among participants in an adenovirus vaccine clinical trial. In terms of immunogenicity, the majority of studies found that the approved vaccines were effective in stimulating the production of neutralizing antibodies against the receptor-binding domains (RBDs) of SARS-CoV-2 (18, 21–24, 28, 29, 36). However, more studies are needed to assess the persistence of immunity after vaccination. There are currently no published systematic reviews or meta-analyses that integrate and evaluate the efficacy and safety of all COVID-19 vaccines. The main aim of this study was to investigate the published literatures in order to evaluate the efficacy, immunogenicity, and safety of the COVID-19 vaccines.

## Methods

### Definitions and Outcomes

The efficacy of a COVID-19 vaccine was defined as the relative reduction in SARS-CoV-2 infection risk following vaccination, as determined by previously published randomized placebo-controlled clinical trials (26, 30, 32, 37). The safety of a COVID-19 vaccine was determined in this study as the health outcome after vaccination under acceptable conditions, as defined by previously published research. This study included both observational, randomized and non-randomized controlled studies. Positive reverse transcriptase PCR (RT-PCR) results for COVID-19 were considered as laboratory-confirmed cases. Published observational studies and randomized and non-randomized controlled trials were selected based on the inclusion criteria. Efficacy was considered statistically significant when the 95% CI for efficacy did not cross 0 for all studies. The reporting of this systematic review was guided by the standards of the Preferred Reporting Items for Systematic Review and Meta-Analysis (PRISMA) statement (38).

### Search Strategy and Selection Criteria

Different electronic websites, databases, and journals, including MEDLINE (through PubMed), EMBASE, Web of Science, Scopus, The Lancet, and The New England Journal of Medicine (NEJM), were searched to detect published articles on the efficacy and safety of the COVID-19 vaccines from December 15, 2019 to May 15, 2021. Preprint repositories such as medRxiv, bioRxiv, SSRN, and AAS Open Research were also searched for related preprint articles. Additionally, the first 20 pages of the Google Scholar search engine were manually screened for relevant articles. The language of the articles that were reviewed was limited to English. This study used the following combinations as search terms: COVID-19, SARS‐CoV‐2, vaccine, safety, efficacy, side effects, effectiveness, clinical trial, observational study, randomized controlled study, mRNA vaccine, adenovirus vector vaccine, subunit vaccine, inactivated vaccine, variants, B.1.1.7, B.1.351, P.1, B.1.526, B.1.429, B.1.617, alpha, beta, gamma, delta, iota, epsilon, China, the USA, the UK, India, Russia, Australia, Brazil, ChAdOx1 nCoV-19, Ad26.COV2.S, mRNA-1273, BNT162b1, BNT162b2, rAd26, rAd5, and MF59-adjuvanted spike glycoprotein-clamp.

As this is an early meat-data analysis based on the available studies published within the last 2 years, it included studies on any strain of SARS-CoV-2. NS and SD evaluated the eligible studies. All included studies were evaluated for quality by NS, KA, and SA. The risk of bias was studied using The Systematic Review Centre for Laboratory Animal Experimentation (SYRCLE) assessment tool (39). The evaluation of SYRCLE consisted of 10 parameters to assess various biases, including attrition bias, selection bias, detection bias, reporting bias, performance bias, and other biases. The measurement of bias was done using possible outcomes for each parameter as yes, no, and unclear, indicating low, high, and unclear risk of bias, respectively (39).

This review excluded studies on the acceptance and challenges of the COVID-19 vaccines, which were not related to natural infection and were not comparable to other studies. The exclusion criteria were as follows: studies that only reported nonspecific outcomes, such as a reduction in the period of illness, mortality, or COVID-19-like illness; studies that did not provide efficacy or safety or immunogenicity data for the COVID-19 vaccines; and non-comparable parallel studies with unspecific clinical outcomes, as unspecific outcomes could lead to unmeasured confounding and complicate the interpretation.

We could not implement seasonal exclusion criteria due to the lack of seasonal studies. We could not also rule out studies that did not report specific variant properties due to lack of data. Safety and side effect studies based on self-reporting were excluded. Safety studies were excluded unless they used systematic sampling of participants using well-defined symptom criteria.

### Inclusion Criteria for Efficacy Studies

Randomized controlled trials published in peer-reviewed journals indexed by PubMed were included in this analysis. Studies with large numbers of participants in which the outcomes were defined as RT-PCR-confirmed cases by following standard guidelines of the WHO were included in this article. We also included studies where the control group received a placebo or a vaccine other than SARS-CoV-2 and studies where the concentrations of the mRNA vaccines were presented in microgram amounts and the adenovirus vector vaccines in 10^10^–10^11^ virus particles.

### Inclusion Criteria for Safety and Immunogenicity Studies

This work included articles that published phase I/phase II/phase III clinical trials of the COVID-19 vaccines in peer-reviewed journals indexed by PubMed. We included studies that measured the severity of the side effects of the COVID-19 vaccines using the WHO guidelines; studies reporting the production of neutralizing antibodies against RBDs within 0–30 days of vaccination; studies reporting the immunogenicity of both the first and second doses of the vaccine; studies in which the control group received placebo or vaccines other than SARS-CoV-2; and studies in which the side effects were monitored and evaluated by experts. Summary data were extracted from published works for all of the studies.

### Statistical Analysis

This study used the Mantel–Haenszel fixed effects method (random effects pooled odds ratios with 95% CI) for three or more randomized controlled trials on similar COVID-19 vaccines. The Breslow–Day statistic was used to assess the homogeneity of the odds ratios. Vaccine efficacy was calculated using the random effects odds ratio. This study applied the accepted statistical vaccine efficacy formula, (1 − odds ratio) × 100, for calculating the pooled odds ratios to establish pooled vaccine efficacy. This study interpreted the protective efficacy point of the vaccine and the CI. We included negative estimations as zero efficacy. Statistical analyses were conducted using SAS version 9.4.

## Results

### Study Analysis and Efficacy of the Vaccines

**Figure 1:**
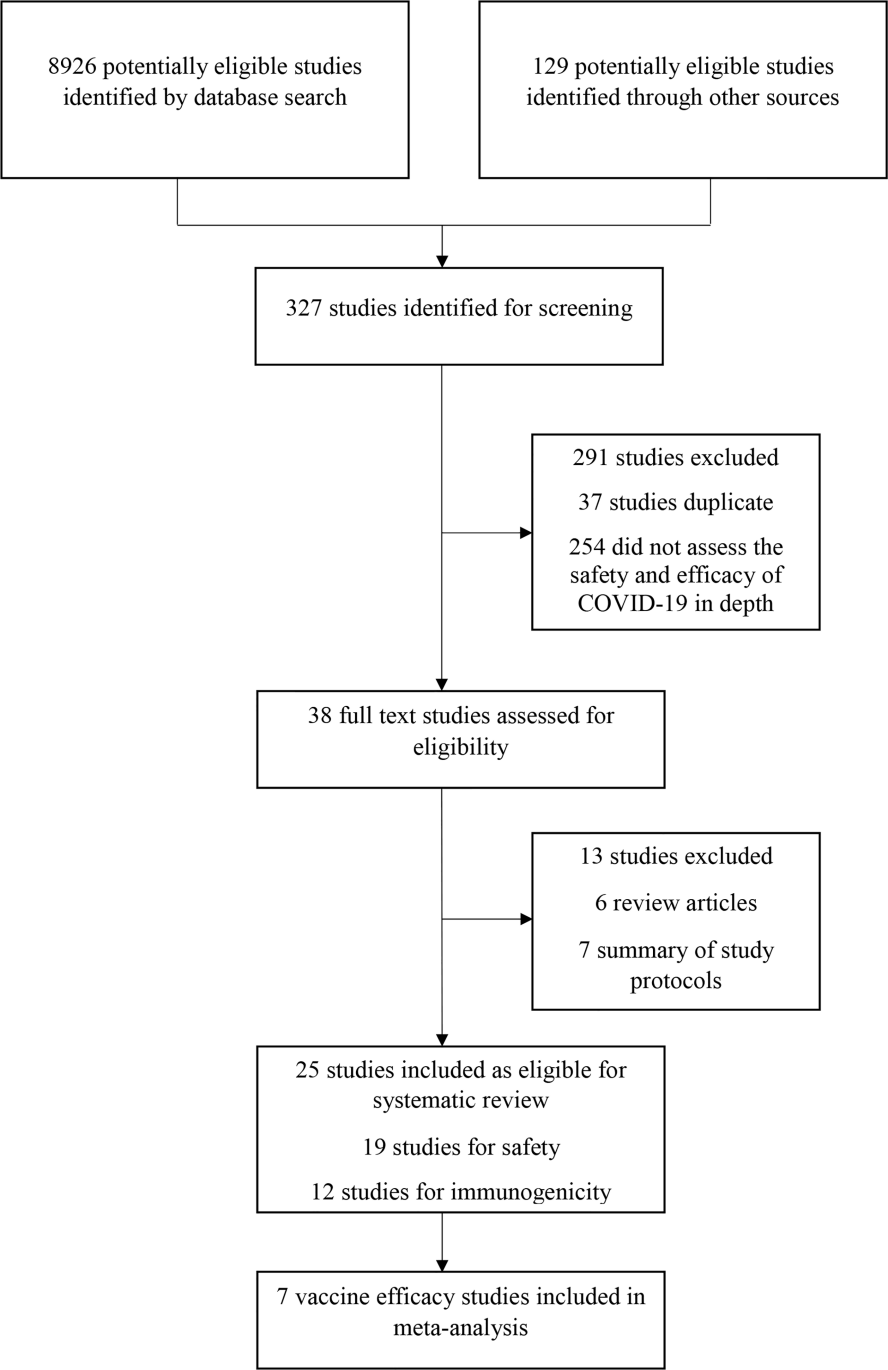
Study selection procedure.

This study found 8,926 research articles on COVID-19 and SARS-CoV-2 vaccines by using the previously mentioned search terms (**Figure 1**). From the 8,926 articles, 327 articles were screened and found eligible for further investigation. After analyzing the abstracts, only 38 (11.6%) studies were included. Based on the inclusion criteria, 25 (65.8%) of the 38 articles were eventually chosen. Of these, 19 (76%) studies were analyzed for evaluating safety, 12 (48%) studies were analyzed for assessing immunogenicity, and 7 (28%) studies were analyzed for evaluating the efficacy of the COVID-19 vaccines. Three studies (12%) were observational, 19 (76%) were randomized controlled trials, and 2 (8%) were non-randomized clinical trials. This review included four different types of COVID-19 vaccines. Studies on the adenovirus vector vaccines (11, 44%) were the most prevalent, followed by studies on the mRNA vaccines (10, 40%), studies on the inactivated virus vaccines (4, 16%), and studies on the recombinant subunit vaccines (2, 8%) (**Table 1**). Clinical trials of the mRNA vaccines (mRNA-1273, BNT162b1, and BNT162b2), adenovirus vector vaccine (ChAdOx1 nCoV-19, non-replicating adenovirus type 5), and inactivated virus vaccine (BBIBP-CorV) began during the first quarter (March/April) of 2020 (24, 26, 27, 30, 32, 33, 37, 40).

**Table 1:** Number of trials of phase I/II/III and observational studies published safety, efficacy and immunogenicity of COVID-19 vaccines.

Vaccine type	No. of published trials	Age groups (years) in trials	No. of doses	Study period
**mRNA vaccines**				
mRNA-1273	2	18 to <65, ≥65, 56–70, ≥70	2	April 2020
				November 2020
BNT162b1	3	18–55, 65–85	2	April 2020
				May 2020
BNT162b2	5	18–55, 65–85, >60, 16–55, >55, 16–39, 40–49, 50–59, 60–69, 70–79, ≥80	2	October 2020
				December 2020
				February 2021
**Adenovirus vector vaccines**				
ChAdOx1 nCoV-19 (AZD1222)	6	18–55, 56–69, ≥70, ≥18, 18–55, >60	2	April–November 2020
				May–August 2020
				May–November 2020
				December 2020–March 2021
Non-replicating adenovirus type 5 (Ad5)-vectored	2	18–29, 30–39, 40–49, 50–60, 18–44, 45–54, ≥55	1	March 2020
				April 2020
Ad26.COV2.S	1	18–59, ≥60	1	September 2020
rAd26 and rAd5 vector-based heterologous prime boost	2	18–30, 31–40, 41–50, 51–60, >60, >18	2	June–August 2020
				September–November 2020
**Inactivated virus vaccines**				
BBIBP-CorV	2	18–59, ≥60	2	April–June 2020
CoronaVac	2	18–59	2	May–Jun 2020
**Subunit vaccines**				
MF59-adjuvanted spike glycoprotein-clamp	1	≥18 to ≤55	2	June–August 2020
NVX-CoV2373	1	18–59	2	May 2020

**Figure 2:**
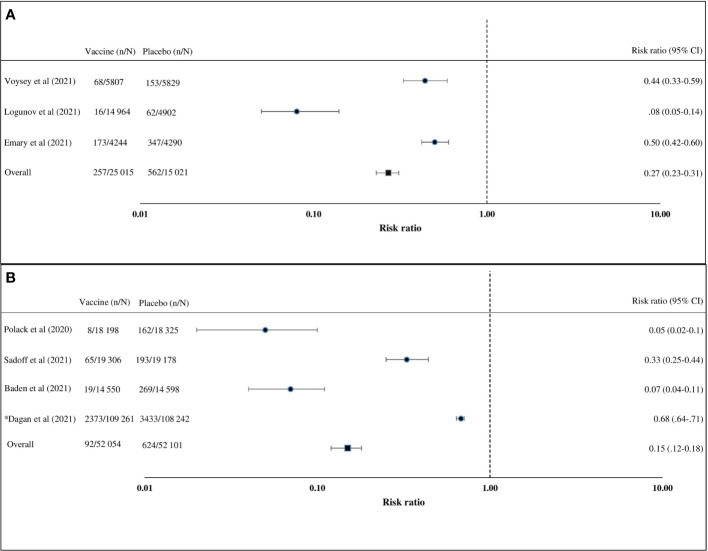
Vaccine efficacy compared with placebo calculated using the Mantel–Haenszel random effects model. **(A)** Adenovirus vector vaccines in participants aged 18 years or above. **(B)** Messenger RNA (mRNA) vaccines in participants aged 16 years or above. Prospective studies (risk ratio) were included in this analyses. *n*, cases of COVID-19; *N*, group size.

Of the seven articles on the efficacy of the COVID-19 vaccine, four (57%) were on the adenovirus vector vaccine and three (43%) on the mRNA vaccines (**Table 2**) (24, 26, 27, 30, 32, 33, 37, 40). Studies on the adenovirus vector vaccines included two articles on the ChAdOx1 nCoV-19 (AZD1222) vaccine, one article on the rAd26 and rAd5 vector-based (Gam-COVID-Vac) vaccine, and one article on the Ad26.COV2.S vaccine. Published articles on the adenovirus vector vaccines reported clinical trials on uninfected healthy persons aged 18 years or above. The adenovirus vector vaccine (Gam-COVID-Vac) was reported to have the highest efficacy (91.6%, 95% CI = 85.6–95.2), followed by ChAdOx1 nCoV-19 (AZD1222) (70.4%, 95% CI = 54.8–80.6) and Ad26.COV2.S (66.1%, 95% CI = 55–74.8) (24, 26, 30, 32, 37). Of the three studies on the efficacy of mRNA vaccines, two were on the BNT162b2 vaccine and one was on the mRNA-1273 vaccine (27, 40, 41). An observational study and a randomized controlled clinical trial were conducted on the BNT162b2 vaccine. About 92% (95% CI = 88–95) efficacy was found in the observational study, while 95% (95% CI = 90.3–97.6) efficacy was established in the randomized controlled trial on the BNT162b2 vaccine (27, 41). One randomized controlled trial on the mRNA-1273 vaccine observed 94.1% (95% CI = 89.3–96.8) efficacy (40). Studies on the BNT162b2 vaccine included uninfected participants aged 16 years or older, and the study on the mRNA-1273 vaccine included uninfected participants aged 18 years or older. Only a few trials have been carried out to determine the efficacy of the COVID-19 vaccines against circulating variants (15, 30, 37). An adenovirus vector vaccine, ChAdOx1 nCoV-19 (AZD1222), was trialed against the variant of concern, 202012/01 (B.1.1.7), and shown to have a lower efficacy than against the non-B.1.1.7 variant (30). Another adenovirus vector vaccine, Ad26.COV2.S, showed similar efficacies against the 20H/501Y.V2 variant and the non-20H/501Y.V2 variant (37). The random effects pooled efficacy for the adenovirus vector vaccine was 73% (95% CI = 69–77) and for the mRNA vaccine was 85% (95% CI = 82–88). An observational study was excluded during the calculation of pooled vaccine efficacy (**Figure 2**). Clinical trials on the mRNA vaccines (mRNA-1273, BNT162b1, and BNT162b2), adenovirus vector vaccines (ChAdOx1 nCoV-19, non-replicating adenovirus type 5, and Ad26.COV2.S), and inactivated virus vaccine (BBIBP-CorV) reported that the vaccines provided protection in about 98%–100% of the recipients against death related to COVID-19 (24, 26, 27, 30, 32, 33, 37, 40).

**Table 2:** Randomized controlled trials and observational studies fulfilling the inclusion criteria for the efficacy of COVID-19 vaccines.

Study	Population	Study type	Country	Ethnicity	Intervention	No. of participants	Controls	Vaccine efficacy (95% CI)
Polack et al. (27)	16-year-olds or older	Randomized controlled trial	Argentina, Brazil, South Africa, United States	White, Black, or African American, Asian, Native American, Multiracial, Hispanic, Native Hawaiian, Not reported	BNT162b2 vaccine; 2 doses	43,548	21,728	95% (90.3–97.6)
Voysey et al. (26)	18-year-olds or older	Randomized controlled trial	UK, Brazil	White, Black, Asian, Mixed, Other	ChAdOx1 nCoV-19 (AZD1222); 2 doses	23,848	12,212	70.4% (54.8–80.6)
Logunov et al. (32)	18-year-olds or older	Randomized controlled trial	Russia	White, Asian, Other	rAd26 and rAd5 vector-based (Gam-COVID-Vac); 2 doses	21,977	5,476	91.6% (85.6–95.2)
Emary et al. (30)*	18-year-olds or older	Randomized controlled trial	England, Wales, Scotland	White	ChAdOx1 nCoV-19 (AZD1222); 2 doses	8,534	4,267	70.4% (43.6–84.5)
Sadoff et al. (37)*	18-year-olds or older	Randomized controlled trial	Latin America, Argentina, Brazil, Chile, Colombia, Mexico, Peru, South Africa, United States	American Indian or Alaskan Native, Indigenous South American, Asian, Black, Native Hawaiian or other Pacific Islander, White, Multiracial	Ad26.COV2.S; 1 dose	39,321	19,691	66.1% (55–74.8)
Baden et al. (40)	18-year-olds or older	Randomized controlled trial	United States	White, Black, or African American, Asian, American Indian or Alaska Native, Native Hawaiian or Other Pacific Islander, Multiracial	mRNA-1273; 2 doses	30,420	15,210	94.1% (89.3–96.8)
Dagan et al. (41)	16-year-olds or older	Observational study	Israel	General Jewish, Arab, Ultra-Orthodox Jewish	BNT162b2; 2 doses	596,618	596,618	92% (88–95)

*Indicated studies including variant alpha (B.1.1.7)/beta (B.1.351)/gamma (P.1).

### Immunogenicity of the COVID-19 Vaccines

Following the inclusion criteria, 12 published studies on the immunogenicity of COVID-19 vaccines were included in this systematic review article. These studies reported successful production of antibodies against the RBDs of SARS-CoV-2 in about >90% of vaccinated study participants. The ability of the COVID-19 vaccines to induce T-cell-mediated immunity among participants was also assessed (**Table 3**). The COVID-19 vaccines, including the non-replicating adenovirus type 5 (Ad5)-vectored vaccine, BNT162b1, Gam-COVID-Vac, mRNA-1273, and NVX-CoV2373, had the capability to induce the immune system to producing from 10^2^ to ≤10^3^ T cells per 1 × 10^5^ peripheral blood mononuclear cells (PBMCs) in the vaccinated population in the first 28 days after vaccination. The safe and effective dose concentrations differed depending on the vaccine type. Among 12 studies, four (33.3%) were on the adenovirus vector vaccine, four (33.3%) were on the mRNA vaccine, two (16.7%) were on the inactivated vaccine, and two studies (16.7%) were on the subunit vaccine. The time it took for vaccine recipients to achieve effective seroconversion varied depending on the vaccine type and the dose concentrations (**Table 3**). Studies on the adenovirus vector vaccine reported that about 10^10^–10^11^ virus particles successfully stimulated the production of neutralizing antibody against RBDs within 0–28 days after vaccination (16–20). Furthermore, studies on the mRNA vaccines found that 25–30 μg doses were able to induce neutralizing antibody production in individuals within 0–28 days after vaccination (21, 23, 26, 36). One study on the subunit vaccine in Australia reported that about 99% (67 of 68) of participants receiving two doses developed neutralizing antibodies within 57 days (29). Another study on the recombinant spike protein nanoparticle vaccine (NVX-CoV2373) also reported that the vaccine was able to induce anti-spike immunoglobulin G (IgG) and neutralization response in vaccinated participants within 28 days after vaccination (22).

**Table 3:** Clinical trials on the immunogenicity of COVID-19 vaccines included in this study.

Study	Vaccine type	Dose concentration	Days after immunological reaction peaked	No. of vaccine recipients	Recipients with ≥4× increase of neutralizing antibody against RBDs (GMT) (%)	Recipients with TNF-α-secreting CD4^+^ and CD8^+^ T cells (%)	Recipients with >10^2^ IFN-γ-expressing cells per 1 × 10^5^ PBMCs (%)	Recipients with IL-2-secreting CD4^+^ and CD8^+^ T cells (%)
**Adenovirus vector vaccines**								
Zhu et al. (19)	Non-replicating adenovirus type 5 (Ad5)-vectored	Middle dose	28	36	94	92	90	92
Zhu et al. (20)	Non-replicating adenovirus type 5 (Ad5)-vectored	1 × 10^11^ viral particles	28	253	97	–	90	–
Folegatti et al. (16)	ChAdOx1 nCoV-19	5 × 10^10^ viral particles	28	35	100	–	–	–
Logunov et al. (17)	Gam-COVID-Vac	10^11^ viral particles	28	20	100	100	–	–
Logunov et al. (17)	Gam-COVID-Vac-Lyo	10^11^ viral particles	28	20	100	100	–	–
Ramasamy et al. (18)	ChAdOx1 nCoV-19	5 × 10^10^ viral particles	28	126	>99	–	–	–
**mRNA vaccines**								
Mulligan et al. (23)	BNT162b1	30 μg	28	12	100	–	–	–
Sahin et al. (36)	BNT162b1	30 μg	28	12	100	100	100	>99
Walsh et al. (28)	BNT162b1	30 μg	28	12	100	–	–	–
Walsh et al. (28)	BNT162b2	30 μg	28	12	100	–	–	–
Anderson et al. (21)	mRNA-1273	25 μg	57	20	100	100	100	100
**Inactivated vaccines**								
Xia et al. (24)	BBIBP-CorV	4 μg	42	24	100	–	–	–
Zhang et al. (35)	CoronaVac	3 μg	28	141	>98	–	–	–
**Subunit vaccines**								
Keech et al. (22)	NVX-CoV2373	25 μg	35	28	100	100	100	100
Chappell et al. (29)	MF59-adjuvanted	15 μg	57	24	100	100	100	100

RBDs, receptor-binding domains; PBMCs, peripheral blood mononuclear cells; GMT, geometric mean titer.

### Safety of COVID-19 Vaccines

Among the studies on the safety of the COVID-19 vaccines, five were on the adenovirus vector vaccine, six were on the mRNA vaccine, two were on the subunit vaccine, and three studies were on the inactivated vaccine (**Table 4**). Local effects including pain, redness, and swelling at the vaccination site and systemic effects such as fever, fatigue, headache, chill, vomiting, diarrhea, nausea, and arthralgia were reported as the side effects of COVID-19 vaccination (16–20, 23, 28, 37). Pain at the injection site was the most common local symptom in the mRNA (29%–85% of participants) and adenovirus vector (0.2%–78% participants) vaccines. Fever (0.2%–95%), fatigue (1%–55%), and headache (0.7%–68%) were the most commonly reported symptoms among those who received adenovirus vector vaccines, whereas fatigue (8.4%–55%) was the most common in those who received mRNA vaccines. Fatigue (30%–40%) and headache (15%–40%) were the most common symptoms among those who received subunit vaccines (21, 22, 24, 27–29, 33–35, 40, 42). According to one adenovirus vector vaccine research, 8% (20 of 253) of those who received the immunization had grade 3 fever (19). Four percent (5,994 of 14,985) of participants in a study had grade 3 pain at the injection site, while 8% (11,988 of 14,985) of participants in a trial reported grade 3 fatigue after receiving the mRNA vaccine (40). In these studies, no deaths associated with COVID-19 vaccines were documented. Furthermore, the published works reported very low frequency of grade 3 and grade 4 local and systematic symptoms in vaccinated participants (21, 22, 24, 27–29, 33–35, 40, 43).

**Table 4:** Published trials and observational studies showing side effects of the COVID-19 vaccines included in this systematic review.

Study	Total no.	Local effects (% of first dose vaccine recipients)			Systemic effects (% of first dose vaccine recipients)							
		Pain	Redness	Swelling	Fever	Fatigue	Headache	Chill	Vomiting	Diarrhea	Nausea	Arthralgia
**Adenovirus vector vaccines**												
Zhu et al. (19)	108	54	4	7	46, 8^a^	44, 2^a^	39	0	2	11	6	17, 1^a^
Zhu et al. (20)	253	57	2	4, <1^a^	32, 8^a^	42, 1^a^	29, 1^a^	0	2	8	8	13, 1^a^
Folegatti et al. (16)	487	67	0	0	70	15	68	56	0	0	20	0
Logunov et al. (17)	9	78	0	0	89	33	67	0	0	11	22	33
Logunov et al. (17)	20	40	0	5	95	55	45	0	0	15	5	20
Ramasamy et al. (18)	98	39	0	0	<1	20, 3^a^	10	10, <1^a^	0	0	<2	<1
Sadoff et al. (37)	21,895	0.2	0.1	0.1	0.2	1	0.7	0	0	0	0.2	0
Menni et al. (33)	345,280	19	4.2	5.5	8.2	21	23	14.7	0	2.2	5.7	11.5
**mRNA vaccines**												
Mulligan et al. (23)	45	85	10	15	21	55, 4^a^	52, 2^a^	42, <1^a^	0	6	0	27
Walsh et al. (28)	84	75	17	21	5	50	0	35	0	0	0	0
Walsh et al. (28)	84	70, <2^a^	<1	<1	6	40	0	31	0	0	0	0
Anderson et al. (21)	40	75	10	18	0	35	30	0	0	0	0	0
Polack et al. (27)	18 860	80	5	6	10	40	35	20	<1	6	0	13
Baden et al. (40)	15 181	70, 4^a^	4	5	0	50	30	0	7	0	8	35
Menni et al. (33)	282 103	29	4	6.4	1.5	8.4	7.8	2.5	0	1.2	2.1	3.2
Monin et al. (42)	40	52	20	7	2	20	15	10	4	3	1	10
**Subunit vaccines**												
Keech et al. (22)	23	42	0	0	0	40	40, 5^a^	0	0	0	5	10
Chappell et al. (29)	24	41	0	0	0	15	30	12	4	11	5	10
**Inactivated virus vaccines**												
Xia et al. (24)	24	29	0	0	4	0	0	0	0	0	0	0
Zhang et al. (35)	24	20	5	5	5	0	5	0	0	5	0	57
Wu et al. (34)	125	12	11	1	3	3	0	0	0	2	1	0

a Grade 3. Grade 1 and 2 side effects increased after the second dose in the groups taking the mRNA and adenovirus vector vaccines. Grades of health effects were defined by previously published works.

## Discussion

This study is one of the early systematic reviews on the efficacy, immunogenicity, and safety of the COVID-19 vaccines. We included specific data from published works using restrictive inclusion criteria. Significant research gap was found on the efficacy of COVID-19 vaccines for multiple age groups and circulating variants (10, 26–28, 30, 32, 37, 40). Of note is that this study represented an integrated overview of the efficacy, safety, and immunogenicity of the COVID-19 vaccines. In this meta-analysis, we detected that the collective vaccine efficacy for the adenovirus vector vaccines was 73% (95% CI = 69–77) and for the mRNA vaccines was 85% (95% CI = 82–88). The efficacy of the COVID-19 vaccines was calculated after vaccination of the final dose.

As of June 30, 2021, we found lack of research and studies on the COVID-19 vaccines in larger populations and against all the circulating variants of SARS-CoV-2 globally. New variants such as the delta variant (B.1.617.2) have the potential to infect people under the age of 16 years and increase the risk of death in young adults (4, 13, 44). Furthermore, variants with significant mutations at the RBDs can possibly escape the immune system of the vaccinated population (13). There are no randomized controlled trials showing the efficacy and safety of the mRNA vaccines, adenovirus vector vaccines, subunit vaccines, and inactivated vaccines in people aged 2–16 years (4, 10). Studies on the efficacy and safety of the inactivated and subunit vaccines in people aged >70 years are also lacking. Moreover, with increasing duration of the pandemic, newer variants are spreading globally (14). Few studies have been conducted on the efficacy of the COVID-19 vaccines against limited circulating variants. The published literatures provided evidence of the good efficacy of the mRNA and adenovirus vector vaccines in people aged over 18 years (26, 27, 30, 32, 40, 41). Efficacy studies on the subunit and inactivated vaccines are not published yet. Evidence from published works and trials on the immunogenicity of the available adenovirus vector and mRNA vaccines suggest that they can provide significant protection against SARS-CoV-2 by producing antibodies against the receptor-binding sites and that T cells mediate protection. However, we could not find studies on the long-term prevalence of antibodies against RBDs or SARS-CoV-2 in the vaccinated population. Furthermore, studies and trials on the subunit and inactivated vaccines also presented evidence of stimulating the production of enough antibodies against RBDs and of T-cell-mediated immunity in vaccinated people (22, 24, 35). Published research papers on the safety of COVID-19 vaccines in participants aged over 18 years found that both local and systemic health effects were aroused at an acceptable frequency. The severity of local and systemic events varied widely from vaccine to vaccine. For any authorized vaccine, grade 1 severity of local and systemic events was the most common, followed by grades 2 and 3. Pain at the injection site was the most common local event in participants who received any type of COVID-19 vaccine, whereas fever, fatigue, and headache were the common systemic effects in those who received the mRNA, adenovirus vector, or the subunit vaccine. Both the first and second doses of the vaccine resulted in adverse health events observed within 0–7 days. After the second dosage of the mRNA and adenovirus vector vaccines, the frequency of adverse health consequences increased in vaccine recipients (25–27, 32, 37, 40).

As of May 10, 2021, only a few systematic reviews and meta-data analyses on the efficacy, immunogenicity, and safety of the COVID-19 vaccines had been published (10, 43, 45). A meta-analysis on the efficacy and safety of the COVID-19 vaccines with limited statistical power has been published (10). Another meta-analysis assessed the immunogenicity and reactogenicity of the COVID-19 vaccines using both human and animal models from phase I/II/III trials (45). In this systematic review and meta-analysis, we reported the collective efficacy, immunogenicity, and safety of the mRNA and adenovirus vector vaccines. Vaccination against COVID-19 has begun in selected populations in various nations using adenovirus vector, mRNA, and inactivated vaccines. The number of randomized clinical trials and studies using COVID-19 vaccines is still limited (24, 26, 27, 30, 32, 37, 40, 41). The COVID-19 pandemic is still ongoing, and a third wave with increased cases and fatalities is possible in countries lacking effective mass vaccination. More randomized controlled trials focusing on mass vaccination and emerging variants can speed up the vaccination and the pandemic mitigation.

This study provides a comprehensive evaluation of the existing literatures on COVID-19 vaccines. We found that the available mRNA, adenovirus, and inactivated vaccines were able to produce significant immune reaction against the RBDs of SARS-CoV-2 among vaccine recipients. Participants generated neutralizing antibodies against RBDs within 30 days after vaccination, after both first and second doses. When compared to other vaccines, including the influenza virus vaccine, the safety of the COVID-19 vaccines—mRNA, adenovirus vector, and inactivated vaccines—was also acceptable (46). Several types of thrombosis (blood clotting), such as deep vein thrombosis (DVT), pulmonary emboli (PE), cerebral venous sinus thrombosis (CVST), and abdominal or arterial clots, have been documented in participants within 4–30 days following vaccination with the adenovirus vector and mRNA vaccines, at a lower frequency (47–50). The vaccination campaign has been continued using available vaccines because these life-threatening incidents are infrequently reported and not well documented yet. Our findings suggest that continued surveillance and randomized clinical studies involving a large number of individuals in various countries on the existing vaccines against emerging variations are essential. Several variants have been shown to induce serious infection in younger adults and children (4, 6, 13). As a result, clinical trials that include a large number of participants under 18 years and emerging variants should be done on a regular basis. Before any government implements a vaccination program, an efficacy research on the subunit and inactivated vaccines should be made available. Given that vaccination has only been in place for less than a year, studies should focus on the long-term efficacy and safety of the currently available COVID-19 vaccines. Available data on the ability of these COVID-19 vaccines to provide sufficient immunity against COVID-19 infection in the vaccinated population support the positive impact of mass vaccination in mitigating the pandemic. However, the major challenge ahead is ensuring rapid and equal dissemination of COVID-19 vaccines to people in both developed and developing countries.

The study’s key strength is that it used high-quality papers published in reputable publications and used strict bias removal procedures to interpret the most precise results. We also applied strict and practical exclusion criteria to remove different biases from this study. Only data from cases that were confirmed using RT-PCR procedures in accordance with the WHO criteria were evaluated in this study, and only randomized clinical studies were included in this meta-analysis for a full evaluation of the efficacy of the COVID-19 vaccines. Previous meta-analysis research on the efficacy of the COVID-19 vaccines only included a small number of studies and samples. However, compared to the previously published meta-analysis, our study found decreased overall efficacy of both the adenovirus vector and mRNA vaccines. We included more studies and samples in our research, which reflected a more precise efficacy than that shown in earlier studies (24, 45).

The main limitation of this study is that, due to the lack of available clinical trials, vaccine efficacy data for the inactivated and subunit vaccines could not be included. Some studies had limited sample sizes, geographic regions, and age groups, resulting in sample bias. This study could not include randomized controlled trials on different emerging variants due to lack of data. Furthermore, due to lack of literature, this study was unable to present the long-term protective impact of COVID-19 vaccines. Data on the efficacy and safety of the COVID-19 vaccines in people with comorbidities and preexisting health conditions, such as pregnancy, were also not included (51).

In conclusion, at present, the available vaccines against COVID-19 are the most effective intervention for containing the pandemic. This study will provide an integrated baseline data on the efficacy and safety of the COVID-19 vaccines for future studies.

## Data Availability Statement

The original contributions presented in the study are included in the article/supplementary material. Further inquiries can be directed to the corresponding author.

## Author Contributions

NS performed systematic and data collection, provided the illustrations, and was a major contributor in writing the manuscript. KA performed critical evaluation, verification of the manuscript, and supported funding. SA performed data analysis and reviewed the article. SD conceptualized the review article and provided oversight, critical evaluation, and verification of the manuscript. All authors contributed to the article and approved the submitted version.

## Funding

This work was partially supported by the Taif University Researchers Supporting Program (project no. TURSP-2020/128).

## Conflict of Interest

The authors declare that the research was conducted in the absence of any commercial or financial relationships that could be construed as a potential conflict of interest.

## Publisher’s Note

All claims expressed in this article are solely those of the authors and do not necessarily represent those of their affiliated organizations, or those of the publisher, the editors and the reviewers. Any product that may be evaluated in this article, or claim that may be made by its manufacturer, is not guaranteed or endorsed by the publisher.
